# Inequality in breast cancer care in a Brazilian capital city: a comparative analysis of narratives

**DOI:** 10.1186/s12939-019-0989-z

**Published:** 2019-06-13

**Authors:** Ana Lúcia Lobo Vianna Cabral, Luana Giatti, Ángel Martínez-Hernáez, Mariângela Leal Cherchiglia

**Affiliations:** 1 Graduate Public Health Program, Medical School, Universidade Federal de Minas Gerais (UFMG), Belo Horizonte, Brazil; 2 Department of Preventive and Social Medicine, Medical School. UFMG, Belo Horizonte, Brazil; 3 Departament d’Antropologia, Filosofia e Treball Social. Medical Anthropology Research Center. Universitat Rovira I Virgili, Tarragona, Spain

**Keywords:** Breast cancer, Qualitative study, Care trajectory, Social inequalities

## Abstract

**Introduction:**

Breast cancer is the leading cause of death by cancer in women in Brazil. Timely access to treatment is a priority for health policy in the country. However, indicators of the disease are not equally distributed between women. Poverty and low levels of schooling associate with late diagnosis, worse prognosis and lower survival.

**Objective:**

To investigate differences between women from different socio-demographic profiles in the breast cancer care trajectory in Belo Horizonte, Brazil.

**Method:**

This is a hermeneutic study through narrative analysis. The selection of the participants was based on data from hospital records of four public and private oncology services in Belo Horizonte, Brazil, according to the following variables: age, levels of schooling, and treatment cost source (Proxy of income): In-depth interviews were performed with 35 women characterized in three profiles: Profile 1 (*n* = 7), age range 51–69 years, schooling ≥15 years and private treatment cost; Profile 2 (*n* = 13), age range 35–58 years, schooling = 11 years and predominantly public treatment costing; Profile 3 (*n* = 15), age range 43–79 years, schooling ≤ 8 years and public treatment cost.

**Results:**

The analysis of the narratives allowed the identification of three main themes (preventive care and first signs/symptoms; search for care and diagnosis of cancer; treatment and perceptions about care received) that highlighted differences between the trajectories, with prejudice to women with characteristics of greater vulnerability (Profile 3).

**Conclusion:**

Although in Brazil the attention to women with breast cancer is guided by principles of equality and equity of care, it is necessary to develop mechanisms to prevent discriminatory practices and that guarantee equality of access to diagnosis and treatment.

## Introduction

Female breast cancer has become the leading cause of cancer death in low and middle-income countries and, therefore, a challenge to their health systems [1]. In Brazil, about 40% of the cases are diagnosed in stages III or IV, and only after the onset of symptoms [2].

Prevention actions and timely access to resources of medium and high complexity for diagnosis and treatment of breast cancer are priorities of public health policy in Brazil [2]. In addition, the Brazilian public health system plays important role in oncological care. Patients diagnosed with breast cancer through private health insurance often migrate to public health system for chemotherapy and radiotherapy, among other procedures [3]. Therefore it is expected that there will be no inequality in access to diagnosis and treatment caused by economic or social disparities. However, breast cancer diagnosis, treatment, and mortality indicators are not equally distributed among the female population. Some characteristics, such as poverty, low schooling, and non-white ethnicity have been associated with late diagnosis, worse prognosis and lower 5-year survival [4, 5].

Differentials in access to treatment for breast cancer according to individual attributes have been previously identified. In a Brazilian capital it was observed that non-white women with less than 8 years of schooling, when compared to white women with 12 years or more of schooling, had a greater probability of waiting > 60 days to start treatment after diagnosis, even when already registered at an oncology service. According to the authors, characteristics of social vulnerability are associated with the production of inequalities even after the access barriers have been overcome [6]. Qualitative studies have contributed to the understanding of women’s behaviors and perceptions towards seeking preventive care [7, 8]. The most frequently investigated population segment is one that presents characteristics of social vulnerability and does not fit into said care, either due to difficulties in scheduling appointments and examinations, or due to behavioral barriers (lack of information, fear, shame, etc.). Given this context, it is necessary to know how women from different social groups perceive their disease process and care trajectory to establish comparisons and investigate differences in the care received.

Through an interpretative analysis of the narratives of women with different sociodemographic profiles of breast cancer diagnosis and treatment in Belo Horizonte, Brazil, this study aimed to investigate differences in their care trajectory and to verify the mechanisms involved in producing these differences.

## Methods

It is a hermeneutic study in which the meanings attributed to experience are explored according to the perspective of those who experienced it [9, 10]. Hermeneutic or interpretive methodology has been used in the field of medical anthropology for the analysis of the cultural and symbolic dimensions of health, disease and care processes [11].

The strategy used to know the experiences of women in breast cancer treatment was the analysis of narratives. In the narrative of lived experience, the sequence of events is established in a coherent way, according to the degree of importance and significance that the narrator attributes to each event [10]. In this way, it is possible to access central aspects of the elaboration of the experience of illness according to specific social contexts [12].

Subject selection was based on data from hospital records of four public and private oncology services in Belo Horizonte, Brazil, according to the following variables: *age, schooling level and treatment cost source* (income Proxy). The search met the following criteria: women undergoing breast cancer treatment diagnosed between 2011 and 2015, older than 18 years, living in the municipality and with valid information on the defined variables. Two hundred fifty records were selected. Based on these data three profiles were characterized: Profile 1 (*N* = 80), women aged 47 to 72 years, predominantly with ≥ 15 years of schooling and exclusively private treatment funding; Profile 2 (*N* = 78) women aged 40 to 71 years, with 11 years of schooling and predominantly public treatment funding; Profile 3 (*N* = 92) women aged 34 to 82 years, with ≤8 of schooling and exclusively public treatment funding. The recruitment of the participants was started from the women in profile 1 followed by profiles 2 and 3. It was finalized when the sufficient number of interviewees in each profile was reached. This number was defined through the technique of conceptual saturation. Of the 250 women initially selected, 57 were contacted by phone; after hearing the explanation of the study objectives, 35 agreed to be interviewed. Of these, seven (7) had characteristics of profile 1, thirteen (13) of profile 2 and fifteen (15) of profile 3.

In-depth interviews [13] were conducted by ALLVC the between April and July, 2016. The following request was made to the women: *I would like you to tell me everything about this episode in your life* [breast cancer]*, from the moment you think it started.* In the narrative about the trajectory, some specific information interested us: habits and perceptions about preventive care; the moment of identification of signs or symptoms (suspected); perceptions about attention received.

Five pilot interviews tested the appropriateness of the guiding question. In addition to the conceptual saturation technique applied to the interviews with each of the profiles, to ensure the validity and reliability of results, we identified and assessed exceptions, compared women in the same profile and between different profiles and contacted some of the respondents by phone to confirm information, according to the Consolidated Criteria for Reporting Qualitative Research (COREQ) [14].

The interviews were transcribed verbatim by ALLVC and four research assistants. The narrative of each subject was identified with the letter P (profile) followed by the profile number (1,2,3) and the respondent’s number (N) of order within the profile. In the next stage of the analysis we aim to identify the elements that, according to Good [10], characterize the construction of the illness narratives, namely: the construction of the plot, which indicates the sequential organization of events and the relation between them; the meanings attributed to illness and the facts and events highlighted in the construction of the narrative; the experience of illness in the interviewee life context . After repeated tapping and reading of the recorded and transcribed material, the researcher proceeded to identify of themes common to the three groups. The product of this work was the construction of an interpretive synthesis of narratives of each profile, structured around the themes identified.

This study is part of the research project “*Mulheres com câncer de mama em Belo Horizonte: perfil, trajetória e representações sobre o cuidado*” [Women with breast cancer in Belo Horizonte: profile, trajectory and representations about care], approved by the Research Ethics Committee of the *Universidade Federal de Minas Gerais* (UFMG), process 48,120,614.3.0000.5149. All ethical precepts were respected. The subjects read and signed the Informed Consent Form before the interview began and had their identity preserved in result presentation.

## Results

### Description of respondents’ profiles

Women in profile 1 were aged between 51 and 69 years, had college-level education and their treatment was privately funded. In most cases, the disease was diagnosed at early stages. In four cases, the suspicion was raised based on screening mammograms. Other characteristics of this profile are presented in Table 1.

**Table 1:** Characterization of women: profile 1

Profile/N of the interviewee	Age	Profession	Detection of suspicious lesion^a^	Staging upon diagnosis^a^	First treatment^b^
P1/01	51	Educator	Mammography	I	Breast conserving surgery
P1/02	51	Teacher/lecturer	Mammography	0	Breast conserving surgery
P1/03	51	Shop assistant	Mammography	I	Breast conserving surgery
P1/04	52	Teacher/lecturer^c^	Mammography	IIB	Breast conserving surgery
P1/05	60	Teacher/lecturer	Nodule	I	Breast conserving surgery
P1/06	65	Civil servant^c^	Nodule	I	Breast conserving surgery
P1/07	69	Educator^c^	Discharge	I	Breast conserving surgery

^a^Data from the RHC/INCA

^b^Information given by the participant. Breast conserving surgery: resection of a breast segment (comprises segmental mastectomy, enlarged lumpectomy and quadrantectomy), with excision of axillary lymph nodes or sentinel lymph node

^c^Retired

Women in profile 2 were aged between 35 and 58 years. They had an intermediate level of schooling (11 years of study) and their cancer care is predominantly funded by the Brazilian public health system (SUS). In this group, there was great variability in their socioeconomic level, and the highest number of advanced cases (Table 2).

**Table 2:** Characterization of women: profile 2

Profile/N of the interviewee	Age	Profession	Detection of suspicious lesion^a^	Staging upon diagnosis^a^	First treatment^b^
P2/01	53	Housewife	Mammography	I	Breast conserving surgery
P2/02	50	Shop assistant/cleaner	Mammography	I	Breast conserving surgery
P2/03	55	Office clerk^c^	Mammography	I	Breast conserving surgery
P2/04	58	Housewife	Mammography	I	Breast conserving surgery
P2/05	41	Housewife	Nodule	IIIB	Neoadjuvant therapy
P2/06	52	Shop assistant	Nodule	IIIB	Neoadjuvant therapy
P2/07	55	Accountancy technician^c^	Nodule	IIA	Breast conserving surgery
P2/08	35	Housewife	Nodule	IIIB	Total mastectomy
P2/09	45	Housewife	Nodule	IV	Neoadjuvant therapy
P2/10	50	Housewife	Nodule	IIIB	Neoadjuvant therapy
P2/11	53	Office clerk	Nodule	IIA	Breast conserving surgery
P2/12	54	Confectioner	Retraction	IIIB	Neoadjuvant therapy
P2/13	55	Office clerk	Retraction	I	Breast conserving surgery

^a^Data from the RHC/INCA

^b^Information given by the participant. Breast conserving surgery: resection of a breast segment (comprises segmental mastectomy, enlarged lumpectomy and quadrantectomy), with excision of axillary lymph nodes or sentinel lymph node

^c^Retired

Women in profile 3 were aged between 43 and 79 years, had < 8 years of schooling and their care was funded by the Brazilian public health system. It is an economically homogeneous group, consisting mainly of house maids, cleaning and janitor service workers, or unemployed workers. This profile had the lowest number of stage 1 cases (Table 3).

**Table 3:** Characterization of women: profile 3

Profile/N of the interviewee	Age	Profession	Detection of suspicious lesion^a^	Staging upon diagnosis^b^	First treatment^c^
P3/01	51	Cleaner	Mammography	IIA	Breast conserving surgery
P3/02	59	Forwarding agent^c^	Mammography	IIIA	Breast conserving surgery
P3/03	67	House maid^c^	Mammography	I	Breast conserving surgery
P3/04	46	Clerical assistant	Mammography	I	Breast conserving surgery
P3/05	66	Pastry cook^d^	Mammography	IIA	Breast conserving surgery
P3/06	59	House maid^c^	Nodule	IIIB	Neoadjuvant therapy
P3/07	69	House maid^c^	Nodule	IIA	Breast conserving surgery
P3/08	70	Clerical assistant^c^	Nodule	IIIC	Neoadjuvant therapy
P3/09	79	House maid^c^	Nodule	IIIA	Total mastectomy
P3/10	65	House maid^c^	Nodule	I	Breast conserving surgery
P3/11	73	Nursing practitioner^c^	Nodule	IIIB	Neoadjuvant therapy
P3/12	43	House maid^d^	Nodule	IIA	Simple mastectomy
P3/13	48	House maid	Nodule	IIIB	Neoadjuvant therapy
P3/14	53	Hairdresser assistant	Nodule	IIIB	Neoadjuvant therapy
P3/15	68	Housewife	Nodule	IIA	Breast conserving surgery

^a^Data from the RHC/INCA

^b^Information given by the participant. Breast conserving surgery: resection of a breast segment (comprises segmental mastectomy, enlarged lumpectomy and quadrantectomy), with excision of axillary lymph nodes or sentinel lymph node

^c^Retired

^d^Unemployed

### Narratives and themes identified

Starting from suspicion (signal/symptom or image), the three groups of women described similar trajectories, mostly determined by the organization of health services, whether public or private: the wait for appointments and examinations, until confirmation of diagnosis and definition of the treatment.

Amidst the diverse accounts, it was possible to find common themes to the three groups. Three of them converged with the purpose of this study and will be subjects of analysis. They are the following: *Preventive care and first signs/symptoms*; *Search for care and cancer diagnosis*; *Treatment and perceptions about care received.*

#### Preventive care and first signs/symptoms

The narratives of women in profile 1 immediately showed their awareness of the need for regular care. They described themselves as concerned with their health, which translated into healthy diets, regular exercise and regular medical checkups, including gynecologic and breast cancer prevention:

*I have always done prevention. Since the age of forty I’ve had mammograms, as per my gynecologist’s orders... I always have and never stopped doing it.* (P1/03)

In four women, the cancer was suspected after a mammogram and in two, the nodules were detected a few months after the examination. The exception was an older woman in the group who had never had a mammogram and had nipple discharge. The reason for not having the test done was a hysterectomy at age 40 years and her claim to not having an active sex life (P1/07).

Among the subjects in profile 2, the narratives showed knowledge about health care and the intent to have regular mammograms - sometimes frustrated by access difficulties.

*We do as the doctor says. Before, I took the exam every year. In the last few years they were spacing out every two years. Then it may be that within these two years the cancer appeared.* (P2/06)

Of the 13 women, eight had regular tests performed, and among these, five had had breast abnormalities detected (nodules or retraction). In this profile, the diagnosis was received as somewhat unexpected. Some narratives show that these women thought cancer should be more likely to occur in those who did not have their tests regularly done. Preventive examinations, such as mammography, are perceived as a way to avoid the disease and not to anticipate the diagnosis.

*I do it every year, exam of prevention of everything, and, I do not know how, my test was positive.* (P2/04)

This belief can be seen in the example given by one of the respondents who, despite a palpable nodule, waited approximately 1 year to see the physician. Since she was on schedule with her mammograms, she did not worry.

*At first, when I discovered the lump, it was a few months away* [for prevention], *I used alternative therapies (for example, crystal) to see if that would oscillate or not, until I did the prevention. So that’s why it took a little bit.* (P2/06)

On the other hand, in women outside the highest-risk age group, the signs and symptoms were underestimated by them or physicians. One of the two youngest women in the group took 2 months to see the physician because she did not believe that a nodule could be serious at her age. As for the other woman who also found a nodule, the fact that she was breastfeeding led the physician to conclude that it was a “milk nodule” and not to order any additional tests. Both were diagnosed with advanced stage disease.

*I was taking a shower and I noticed [the lump]. So I went to the doctor at the health center. But at the time I was breastfeeding and the doctor told me it was a milk nodule. I was 33 at the time he did not ask for mammography. He still said: “mammography is from the age of 40”.* (P2/08)

Post-cancer changes in habits such as quitting smoking and adopting more selective diets were common even if, in the latter case, it increased the burden on the household budget. In profile 2, as in profile 1, the cancer is perceived as a serious disease, but which does not necessarily result in death.

In the profile 3, information about the previous behavior of care, such as routine consultations with gynecologist and mammography, appeared spontaneously in only three interviews. Screening for cervical cancer was more common than for breast cancer. In most interviews the narrative started with the respondent reporting some sign of the disease.

*A lump appeared in my breast, you know? Then, from time to time I would pass the hand to see if it was growing or not, right? Then, after a while, I said, “Oh, that’s nothing.” I let it stay*. (P3/09)

None cited the clinical breast examination that should be performed during the medical appointment. Before falling ill, five women had never done mammography, six did once or twice and four had done routinely.

*No, I did not. The doctor did not ask [..] I decided to ask her. (*P3/03)

Those who rarely or never had the test done did not believe they could ever have the disease. Eight of the respondents in profile 3 had worked or were still working as house maids (as employees or self-employed) and, despite living with other women who regularly had their annual screening mammograms - i.e. the employers - did not find it important to have the exam done, since they did not feel sick.

*No, I never did* [mammography*]. I did not feel anything, I had nothing.* (P3/09)

The five women whose lesions were suspected based on mammograms had the test done while they were on vacations, unemployed or retired.

*[..] Just working, working. And he did not look after me, because he had to work to raise children. I raised 3 children by myself, and God. I did not have much time. When I stopped [..] a motorcycle hit me [..] I could not do anything. So ... I said: “Well, since I cannot work anymore, I’m going to do exams”.* (P3/05)

On the other hand, representations of the disease associated with death, mutilation and disability contributed to the non-adherence to breast cancer prevention practices.

*When it* [lump] *appeared, I must have been in my forties.. “I thought: if I speak, they will want to take my breast off”..I was scared, right?...and I kept quiet, I did not even tell anyone at home!* (P3/09)

#### Search for care and cancer diagnosis

After the suspicion was established, all the women in profile 1 had immediate access to a physician.

*It had been two months since I’d had the mammogram [...] and there was this lump and I called the gynecologist at the time... and he said .. “come over tomorrow”.* (P1/05)

In this case, after an inconclusive biopsy, the physician reassured her saying that it was not a significant finding. She was not happy, and sought a mastologist who diagnosed her with stage I cancer. In all cases in this profile, the surprise and fear of being diagnosed were minimized by comprehensive approaches of receptive professionals.

*She said, “Let’s do this surgery. You’ll be fine.” I asked, “But, doctor, I’m not going to die,?” “No! you’ll be fine!” She gave me a security, a strength, you know?* (P1/06)

In profile 2, the concomitant use of public and private health services was frequent. Primary care appointment, examinations and, in some cases, surgeries were funded by private health insurances, but for high-cost treatments (chemotherapy, radiation therapy and hormone therapy), most of the women resorted to the SUS.

*But the consultations, all the examinations I do for the health plan and the surgeries as well, all I did for the health plan. He* [the doctor] *explained to me that the treatment is very expensive* [and said] *“I’ll give you a referral for the SUS”.* (P2/09)

The exception was a woman who used the public network for some tests, but who was submitted to treatment through private health insurance (P2/01). Among the 13 women, 10 reported delays at some point along the way. Five were blamed on the public health system, two on private health insurances and three were blamed on the patient themselves. In addition, other problems were reported: difficulties in access to mammography; the need to repeat mammography due to poor quality or lost results and exams scheduled on days when the service was not open. In this group, personal relationships (friends or relatives working in the health sector) were constantly used to work around delays in the health care system,

*It took a long time to schedule, almost a month. When [..] I went to get my exam, they had lost my exam. [..] They said: “We’re going to set another date for you to come and do it again.” [..] scheduled for a Saturday afternoon, and it does not work there on Saturday afternoon.* (P2/10)

In Profile 3, ten of the 15 women sought health care because they had found a breast nodule. Some postponed the search because they did not think it was important. Six women blamed the health care systems for the delayed diagnosis. In five cases, it was the women themselves who took too long to seek care. The greatest obstacles to access were found in primary care due to high turnover or lack of physicians in Primary Care Units, or impediments to the performance of mammograms and other tests. Four women reported that the initiative to request a mammogram came from themselves. In one of the cases, she had to insist, because the physician said “it was not necessary” and a moist heat compress would be enough to “dissolve the nodule”.

*I went to do the prevention. I spoke to the doctor who had a lump in his chest. I told her I’d hit my chest. Then she looked and prescribed hot water compress. I did, but I kept the lump in the same place. So I went back there and asked to do the mammography, she said, “No need, but if you want, by conscience, I’ll pass you by.” Then she asked for the mammography.* (P3/15)

Another respondent reported that a breast abscess was diagnosed as a furuncle; after 1 year she was diagnosed with stage IIIB cancer (P3/14).

*Then one day I was taking a shower and my daughter said: “Mom, what’s that on your breast?” “Ah... that, the doctor said it is [...] an ingrown boil”.* (P3/14)

Difficulties scheduling return visits to show test results were also reported. In the case of complementary examinations, such as breast ultrasound, among others, the long waiting list of the public health system led five of the women to pay out-of-pocket for the test, often with the help of family members or people in their church.

*The SUS did not take the exam. It was an expensive [...] exam. The church people who gave me the money and I paid*. (P3/06)

In this profile, another frequent complaint is segmented care. This was observed in cases where, for example, the primary care physician referred the patient to the mastologist who, in turn, referred to another service without, however, informing the primary care team. There is no professional to accompany the woman in the process as a whole, which creates the sense of lack of reference.

#### Treatment and perceptions about care received

Women in profile 1 are more active in the making of treatment decisions. They challenge medical recommendations, they may or may not accept the treatment prescribed, and they choose their physicians. All that is facilitated by the possibility of choosing among the health insurance network or private professionals. Their patient/doctor relationship is horizontal and they have easy access and talk about their physicians in a friendly and informal manner.

*The physician was completely delighted to talk to someone that could easily understand anything he said!* (P1/05)

They seek information about the disease and treatment and, in addition to the mastologist and oncologist, they see a network of different specialists: psychologists, nutrition specialists, and acupuncturists, among others. They strive not to let the treatment have a central role in their lives, and try to reconcile it with their everyday activities, such as work, house chores, education, etc. In some cases, surgeries were planned taking into account vacation time and other commitments. This is possible when the disease is diagnosed at early stages, and requires less urgent and aggressive interventions. Chemotherapy treatment, received by three of the respondents, was considered by one of them as the worst moment of her cancer trajectory. Effects such as hair loss seem to have bothered family members more than the patients themselves, who were clear about the circumstantial and transient nature of these events.

In profile 2, except for one (P2/01), all patients were treated in the public health system. The criterion to define the health care unit is availability or, when possible, proximity to the patient’s residence. Although the possibility of choice is reduced, respondents demonstrated trust the professionals in charge of their care. Patient rarely have any contact with their doctors outside the office. Delays of up to 2 months between diagnosis and onset of treatment were experienced by three women, but the evaluation of the treatment received in the public health system was positive in most cases.

*Well...it takes forever, right? I guess if I would have started sooner, at the beginning...I would have been spared the chemotherapy. But...we have to wait, right?* (P2/04)

*Whenever people ask me about SUS treatment, I say that I have nothing to complain about, I have always been very well taken care of.* (P2/09)

Like women in profile 1, those in profile 2 sought information about the disease and treatment. However they did not challenge medical recommendations. In this group, 11 women received neoadjuvant or adjuvant chemotherapy and, similarly to those in profile 1, the adverse effects of treatment, such as malaise and hair loss, were not relevant. Breast reconstruction is not a major concern in this group, either because they are clear about the right to plastic surgery or because the intervention was not so extensive as to justify the procedure. In both profiles, 2 and 1, anxiety after treatment is constant, as if the cancer were lurking, just waiting for an opportunity to reappear.

*[..] Because, whatever you feel, you think cancer is coming back.* (P1/06)

For the interviewees belonging to these two profiles, the differences between the care received through private health insurance and the care received through the public health system, in hospitals that serve patients of both systems were described as an unjust and disturbing reality.

*There are two machines, when one broke down [..] people from the public health system were removed from the process. But not us: the people with health insurance. We continued with the other machine*. (P1/02)

In profile 3, all women received treatment funded by the public health system. After the diagnosis, the interval to initiate treatment was considered short by most women. However, the definition of short and long interval is somewhat relative. One respondent believes that waiting 6 months for surgery, as her first treatment, was not too long (P3/05).

*It did not take that long to the surgery. About 6 months* [after diagnosis]. (P3/05)

Another considered the three-month wait for neoadjuvant chemotherapy (P3/06) to be short. The benchmark for evaluating the waiting time was the frequent waits of more than 1 year for some tests and appointments.

This impression of the waiting time being short is also related to the feeling that treatment is guaranteed. An after all initial difficulty until diagnosis, finally being linked to an oncology unit, regardless of the time it takes to start treatment, is seen almost as a victory.

[After all] *in the end gave everything right, thank God and that’s fine*. *(P*3/13)

The malaise caused by chemotherapy followed by hair loss and body changes after surgery - whether mastectomy or not - are hard to accept, because they make the disease much more real. With no pain or changes in their everyday life, after the scare of the diagnosis and until treatment starts, the disease is somewhat invisible to these women. In addition, the chemotherapy days - regimens in this group were longer - are marked by long distances traveled and long hospital stays, from early morning to late afternoon, sometimes without proper meals.

*I arrived at the hospital at six o’clock in the morning and took a long time to be seen. The problem is just this: the delay. You spend all day there [..] sometimes I did not eat, because I did not have money* (P3/13)

Due to the bus fare, the poorest women reported going to treatment alone without companions. Upon returning to their homes, some said they did not feel well during the bus trip and were helped by strangers. Although radiation therapy was considered an “easy treatment”, broken machines caused some women to return home without treatment, multiple times. Breast reconstruction with implants does not seem to be a priority for physicians who, in many occasions, did not even mention this subject. The women themselves seemed to believe that this is something superfluous and they had no right to want that.

*The doctor told me that she was going to do it* [reconstruction of the breast], *she promised ... she said she would follow me ... but I never saw her again after the day of the surgery. Not once. It is the absolute worst feeling. We’re missing a piece... and we think...”Man! This is not how it was supposed to be! “ ...* [It would make] *so embarrassed if he said* [the doctor]: *“At this age and still worried about reconstruction?”* (P3/05)

Some attempted to compensate for their frustration, as well as other negative situations, by expressing their gratitude for having received treatment.

The sequelae of axillary lymph node dissection - swelling, difficulty moving and arm numbness - worried some women because they felt they could not work properly. Unlike profiles 1 and 2, the possibility of relapse was mentioned by one woman in profile 3. For most, the end of treatment is interpreted as being cured. All narratives in this group describe, at some point in their trajectory, rough or inappropriate attitudes of some healthcare professional, be it the physician, nurse, or receptionist.

*Then the doctor looked at me and said: “You need surgery.” And my daughter said: - “But, what about her breast?” And she was pretty rude: “Well, I can’t say for sure, but sometimes we have to cut it all out! And if she doesn’t want the surgery, fine...it’s not like I care”. Then she shrugged and left.* (P3/11)

Some of them reported the tension caused by these attitudes, while others did not seem to recognize, despite reporting, apparently inadequate professional conducts, such as paying for medical appointment to speed up the process, even after being enrolled in the public health system care line (P3/11).

*My sister-in-law said: there is a doctor at Hospital X (public hospital), a very good doctor. She is a surgeon and a mastologist. There she charges $150. My husband said: “we’re going to pack the money*” (P3/11)

At all levels of care, interactions are much more frequent with nurses and nursing technicians. Relationships with physicians are distant and formal.

## Discussion

This study compared the breast cancer care trajectories, in a Brazilian capital city, of three different women profiles, defined by age, level of schooling and treatment funding (income proxy).

The main results suggested that the differences found in breast cancer care among the participants may be related to social inequalities, i.e. those that, when associated with individual characteristics such as schooling, income, ethnicity and others, put certain groups at a disadvantage compared to others [15].

Directly or indirectly, these inequalities apparently result in lower-quality care to women of greater vulnerability: asymmetrical relationships with healthcare professionals and services, resulting in negligent and discriminatory service; priority in providing services to private health insurance users to the detriment of public health system users in health units accredited by the public health network and that serve these two groups; difficulties in the course of treatment, such as long and uncomfortable trips in public transport, no companions and no proper meals on chemotherapy days; precarious employment conditions, which hinder the search for preventive care and create lack of confidence due to the risk of unemployment after treatment.

Regarding the preventive care behavior, the narratives of each profile confirmed the findings from the literature: women of higher income and schooling were more likely to perform routine exams than women of lower income and schooling [16]; adherence to screening mammography decreased as age increased (> 69 years) [17, 18]; among younger women, not considering the possibility of having the disease was a factor - attributable to both patients and physicians, which might have contributed to late diagnosis of the disease [19].

Women in profiles 1 and 2, in search of a plausible explanation for the disease, maintained constant vigilance over their own body. Preventive care recommendations from physicians, government guidelines or the media, seem to remit to the individual the responsibility for a possible occurrence of the disease. After treatment, the risk of recurrence exacerbated this self-vigilance in such a way that daily activities were re-evaluated considering the following question: “will this favor cancer or not”? [20].

Castiel [21] drew attention to the “*blaming the victim”* mechanism created by coercive forms of controlling health-related behaviors, based on the autonomy argument. This perspective does not take into account the unpredictability of some health issues, nor the difficulties experienced by people seeking care [22].

Among women in Profile 3, recommendations about preventive exams and how to get them were not ignored. Differently from women in profiles 1 and 2, such practices were not top priorities in their life contexts. According to the literature, low-income working women and those who are the heads of the households tend to use health services less than those living in male-headed households [22]. Moreover, activities lacking social protection, such as informal work (odd jobs) or work performed by day laborers, hamper the regular use of health services [23]. The opening hours of primary care units, which coincide with women’s work hours, are another obstacle. In this context, the search for medical care will take place imperatively, in borderline situations, − pain or any sign of abnormality - which would increase, in theory, the probability of a late diagnosis.

The reasonable waiting time for consultations and examinations among women with private health insurance contrasts with the difficulties faced by users who exclusively used the public system. Fragilities of the public system at the primary and specialty levels encouraged the search for alternatives in the private system. The public-private mix, narrated by women in Profile 2, was an artifice used to overcome the delay in scheduling appointments and examinations. In these cases, vulnerable groups are the most penalized, because with all the obstacles of the public system and without private health insurance, they resorted to out-of-pocket payment to expedite the diagnosis [24]. Facilitating access through personal relationships with healthcare workers was another strategy present in the narratives and previously identified by other authors [25]. It is important to emphasize that the use of parallel and informal systems make it difficult for the patient to link with the team and for the coordination of care which are fundamental roles of primary care [26].

From the perspective of critical medical anthropology, one of the characteristics of the hegemonic biomedical model is to exclude from the “care sphere patients themselves, their biography, their local world and also their social conditions and existence materials” [11]. The disease rather than the patient is the priority of medicine. This model implies an asymmetry in the relationship between physicians and patients, where the former, unilaterally and without prior negotiation, define the rules of treatment [27].

Among the study subjects, these assumptions were shown in the reports of treatment protocols being applied without any dialogue, which creates anxiety and fear. However, women in profile 1, and some in profile 2, exercised their right to information about the treatment processes and the choice to receive them or not. This appropriation of the treatment process is enabled by an active interaction with physicians and other healthcare professionals, which seemed to contribute to decrease the impact of the treatment phase [28].

There is evidence in the literature that good doctor-patient communication positively influences emotional health, functional status, and pain control, but for improved communication to occur there needs to be a “shift in the balance of power between physician and patient” [29].

In the light of these observations, the narratives of women in profile 3 about ironic or disrespectful behaviors of healthcare professionals along the way, especially physicians, may suggest differences in the relationships with different patient profiles. It may be assumed that, in some cases, the greater the difference in social position between physicians and patients, the more distant and less empathic the relationship between them. And the opposite is also true: relationships between physicians and women of similar levels of schooling and income entails more balanced power relations, since both sides have attributes - or symbolic capital - that give them prominent social positions [27].

Studies with healthcare professionals demonstrated the existence of unintentional discriminatory approaches, rooted in negative cultural stereotypes that fall on certain social groups and result in poorer quality of care [28]. Thus, a study conducted in a large Brazilian metropolitan area, women and the poor were more likely to report experiencing discrimination in the search for health care [30]. Moreover, in the South region of the country, low socioeconomic status and ethnicity were associated with perceived discrimination in health care services [31].

Farmer [32] uses the term *structural violence*, originally coined by Galtung [33], to describe attacks on human dignity, reproduced by social structures and their practices predominantly based on historically determined inequalities. According to the author,

*“Social inequalities based on race or ethnicity, gender, beliefs and, above all, social class, are the driving force behind most human rights violations. In other words, violence against individuals is generally embedded in a deep-rooted structural violence”* [32].

The perspective of structural violence allows us to think about discrimination in health care not only as a result of individual prejudice, but also as a product of fundamentally unequal social and economic structures that not only allow, but also naturalize behaviors, such as those observed in this study.

The perception of some women about the differentiated care provided to public patients in private units contracted by the public system which, in face of limited resources, prioritize the provision of services to health insurance patients is evidence of an institutionalized discriminatory practice. A study about the public-private mix in Brazil draws attention to inequities resulting from “inequalities in the supply, access and use of services and the behavior of professionals” between insured and uninsured patients in private units contracted by the Brazilian public health system [34]. In the present study, it is noteworthy that none of the participants with treatment funded by the public system mentioned these disparities in care. Studies on how to measure discrimination stated that, since discriminatory acts are increasingly veiled (whether by law enforcement or social surveillance over politically incorrect behaviors), discrimination is often overlooked by the target [35]. On the other hand, this denial can be a self-protection mechanism of people for whom “recognizing discrimination is like experiencing again the degrading experience” [27].

Adding to this logic, and based on the tolerance seen in women in profile 3 regarding the adversities experienced in their care trajectory, one may suppose that, at the start of treatment, after feeling threatened with being deprived of their right to care, they are grateful as if they were in the receiving end of a huge favor. This effect seems to be fed by and, at the same time, feed the perception of “beneficence” that some professionals have of their work, and which ends up clouding the patient’s awareness of their right to health care [27].

Other dimensions of inequality in health care go beyond the limits of health services and affect the quality of life of women, with devastating consequences. This is the case of mutilating surgeries, as well as omission or denial of the right to breast reconstruction, and other consequences of professional negligence and institutional ineptitude, to which the concept of structural violence can and should be applied to bring such practices to light as a first step towards overcoming them and achieving social justice.

Some limitations of this study require consideration. The respondents in this study do not represent the totality of women on treatment for breast cancer in the city. Information on the itineraries and clinical details of the treatment - except for cancer staging upon diagnosis - were obtained from the participants’ narratives. Therefore, biases are expected and considered important, since they show how respondents perceived the reality they lived in.

## Conclusion

The analysis of the narratives provided clues in pointing out situations that resulted in the loss of quality of attention to women of greater vulnerability and could be understood as discriminatory acts, such as negligent and disrespectful behaviors of health professionals in dealing with the patient.

Other important problems related to the breast cancer care have been identified: precarious labor ties that make it difficult to seek preventive care, suffering during treatment caused by material and immaterial deprivation, lack of basic social support in post-treatment, among others.

Taken together, the findings of this study refer to a citizenship problem that, for vulnerable groups, appears to be a distant and abstract concept. These are issues that should be addressed in future studies to support the formulation of policies and strategies that meet the needs of care of women from different social groups, from prevention to post-treatment of breast cancer, so that social inequalities do not result in inequalities of attention.

Finally, we hope that this study contributes to the qualification of breast cancer care in Brazil. The results presented here reflect the reality of a country that ages - and falls ill - without having solved its main challenge: social inequalities.

## Data Availability

The de-identified interview transcripts and spreadsheets collected, used and analyzed during the current study are available from the corresponding author on reasonable request.

## Abbreviations

N: Number of order within the profile
P: Profile
RHC/INCA: Cancer Hospital Record of the National Cancer Institute database
SUS: Brazilian public health system
UFMG: Universidade Federal de Minas Gerais

## References

[1] Barbosa IR, Costa IDCC, Pérez MMB, de Souza DLB (2015). As Iniquidades sociais e as disparidades na mortalidade por câncer relativo ao gênero. Revista Ciência Plural.

[2] Instituto Nacional de Câncer José Alencar Gomes da Silva. A situação do câncer de mama no Brasil: síntese de dados dos sistemas de informação. / Instituto Nacional de Câncer José Alencar Gomes da Silva. – Rio de Janeiro: INCA; 2019. Available in: https://www.inca.gov.br/sites/ufu.sti.inca.local/files//media/document//a_situacao_ca_mama_brasil_2019.pdf.

[3] Paim J, Travassos C, Almeida C, Bahia L, Macinko J. The Brazilian health system: history, advances, and challenges. Lancet. 2011;377(9779):1778–97. 10.1016/S0140-6736(11)60054-8 [Acesso em: 29 Mar 2019]. 10.1016/S0140-6736(11)60054-8 21561655

[4] Jassem J, Ozmen V, Bacanu F, Drobniene M, Eglitis J, Lakshmaiah KC (2014). Delays in diagnosis and treatment of breast cancer: a multinational analysis. Eur J Pub Health.

[5] Rezende MCR, Koch HA, Figueiredo JA, Thuler LCS (2009). Causas do retardo na confirmação diagnóstica de lesões mamárias em mulheres atendidas em um centro de referência do Sistema Único de Saúde no Rio de Janeiro. Rev Bras Ginecol Obstet.

[6] Cabral ALLV, Giatti L, Casale C, Cherchiglia ML. Social vulnerability and breast cancer: differentials in the interval between diagnosis and treatment of women with different sociodemographic profiles. Ciênc. saúde coletiva [Internet]. 2019 Feb [cited 2019 May 26] ; 24( 2 ):613–22. Available from: http://www.scielo.br/scielo.php?script=sci_arttext&pid=S1413-81232019000200613&lng=en&nrm=iso&tlng=en. 10.1590/1413-81232018242.31672016 30726393

[7] Nonzee NJ, Ragas DM, Ha Luu T, Phisuthikul AM, Tom L, Dong X, Simon MA (2015). Delays in cancer care among low-income minorities despite access. J Women's Health.

[8] Granek L, Fitzgerald B, Fergus K, Clemons M, Heisey R (2012). Travelling on parallel tracks: patient and physician perspectives on why women delay seeking care for breast cancer symptoms. Can Oncol Nurs J.

[9] Clifford G (1989). A interpretação das culturas.

[10] Good BJ. Medicina, racionalidad y experiencia: una perspectiva antropológica. Barcelona: Edicions Bellaterra; 2003.

[11] Martínez-Hernáez A (2008). Antropología médica: teorías sobre la cultura, el poder y la enfermedad.

[12] Nunes ED, Castellanos MEP, Barros NF. A experiência com a doença: da entrevista à narrativa. Physis: Revista de Saúde Coletiva. 2010:20;1341-56.

[13] TMF H (1987). A entrevista. Metodologias Qualitativas na Sociologia.

[14] Tong A, Sainsbury P, Craig J (2007). Consolidated criteria for reporting qualitative research (COREQ): a 32-item checklist for interviews and focus groups. Int J Qual Health Care.

[15] Barata RB. Como e por que as desigualdades sociais fazem mal à saúde. Rio de Janeiro: Editora Fiocruz; 2009.

[16] Oliveira EXG, Pinheiro RS, Melo ECP, Carvalho MS (2011). Condicionantes socioeconômicos e geográficos do acesso à mamografia no Brasil, 2003-2008. Cien Saude Colet.

[17] Santos GDD, RYS C (2011). O conhecimento sobre o câncer de mama e a mamografia das mulheres idosas frequentadoras de centros de convivência em São Paulo (SP, Brasil). Ciên Saúde Colet.

[18] Borges ZDS, Wehrmeister FC, Gomes AP, Gonçalves H (2016). Exame clínico das mamas e mamografia: desigualdades nas regiões Sul e Nordeste do Brasil. Rev Bras Epidemiol.

[19] Montella M, Crispo A, D'aiuto G, De Marco M, De Bellis G, Fabbrocin G (2001). Determinant factors for diagnostic delay in operable breast cancer patients. Eur J Cancer Prev.

[20] Sumalla EC, Ochoa C, Blanco I (2013). «Pero…¿ Estoy curada?». Narración de restitucíon y discurso biomédico em câncer de mama. Evidencias y narrativas en la atención sanitaria Una perspectiva antropológica, Tarragona/Portoalegre, Publicacions de la URV y Rede Unida.

[21] Castiel LD, Dardet CA. A saúde persecutória: os limites da responsabilidade. Rio de Janeiro: Editora Fiocruz; 2007.

[22] Travassos C, Viacava F, Pinheiro R, Brito A (2002). Utilização dos serviços de saúde no Brasil: gênero, características familiares e condição social. Rev Panam Salud Publica.

[23] Miquilin IDOC, Marín-León L, Monteiro MI, Corrêa Filho HR. Desigualdades no acesso e uso dos serviços de saúde entre trabalhadores informais e desempregados: análise da PNAD 2008, Brasil. Cad Saúde Pública. 2013;29:1392-1406. 10.1590/s0102-311x2013000700013 23843006

[24] Göttems LBD, Santos NRC, Souza SFO, Morais TCP, Santana JA (2012). Análise da rede de atenção ao câncer de colo uterino a partir da trajetória de usuárias no Distrito Federal-BR. Rev Eletr Gestão Saúde.

[25] Rangel G, Dias de Lima L, Vargas EP (2015). Condicionantes do diagnóstico tardio do câncer cervical na ótica das mulheres atendidas no Inca. Saúde Debate.

[26] Almeida PF, Marin J, Casotti E (2017). Estratégias para consolidação da coordenação do cuidado pela atenção básica. Trabalho, Educação e Saúde.

[27] Fleury S, Bicudo V, Rangel G (2013). Reacciones a la violencia institucional: estrategias de los pacientes frente al contraderecho a la salud en Brasil. Salud Colectiva.

[28] Aronson J, Burgess D, Phelan SM, Juarez L (2013). Unhealthy interactions: the role of stereotype threat in health disparities. Am J Public Health.

[29] Stewart MA (1995). Effective physician-patient communication and health outcomes: a review. CMAJ: Can Med Assoc J.

[30] Macinko J, Mullachery P, Proietti FA, Lima-Costa MF. Who experiences discrimination in Brazil? Evidence from a large metropolitan region. Int J Equity Health. 2012;11(1):80. 10.1186/1475-9276-11-80. 10.1186/1475-9276-11-80 PMC3542078 23249451

[31] Baumgarten A, Peron B, Bastos JL, Toassi RFC, Hilgert JB, Hugo FN (2015). Experiências de discriminação relacionadas aos serviços de saúde: análise exploratória em duas capitais do Sul do Brasil. Epidemiologia Serviços Saúde.

[32] Farmer P (2005). Pathologies of power: health, human rights, and the new war on the poor.

[33] Galtung J (1969). Violence, peace, and peace research. J Peace Res.

[34] Santos IS (2011). Evidência sobre o mix público-privado em países com cobertura duplicada: agravamento das iniquidades e da segmentação em sistemas nacionais de saúde. Revista Ciência Saúde Coletiva.

[35] Pager D (2006). Medir a discriminação. Tempo Social.

